# Outcomes of End-User Testing of a Care Coordination Mobile App With Families of Children With Special Health Care Needs: Simulation Study

**DOI:** 10.2196/43993

**Published:** 2023-08-28

**Authors:** Willis Wong, David Ming, Sara Pateras, Casey Holmes Fee, Cara Coleman, Michael Docktor, Nirmish Shah, Richard Antonelli

**Affiliations:** 1 Duke University School of Medicine Durham, NC United States; 2 Boston Children's Hospital Boston, MA United States; 3 Family Voices Concord, MA United States; 4 Department of Accountable Care and Clinical Integration Boston Children's Hospital Boston, MA United States; 5 Department of Pediatrics Harvard Medical School Boston, MA United States

**Keywords:** mobile health, mHealth, complex care, care coordination, digital health tools, simulation, family-centered design, user-centered design, participatory design, co-design

## Abstract

**Background:**

Care for children with special health care needs relies on a network of providers who work to address the medical, behavioral, developmental, educational, social, and economic needs of the child and their family. Family-directed, manually created visual depictions of care team composition (ie, care mapping) and detailed note-taking curated by caregivers (eg, care binders) have been shown to enhance care coordination for families of these children, but they are difficult to implement in clinical settings owing to a lack of integration with electronic health records and limited visibility of family-generated insights for care providers. Caremap is an electronic health record–integrated digital personal health record mobile app designed to integrate the benefits of care mapping and care binders. Currently, there is sparse literature describing end-user participation in the co-design of digital health tools. In this paper, we describe a project that evaluated the usability and proof of concept of the Caremap app through end-user simulation.

**Objective:**

This study aimed to conduct proof-of-concept testing of the Caremap app to coordinate care for children with special health care needs and explore early end-user engagement in simulation testing. The specific aims included engaging end users in app co-design via app simulation, evaluating the usability of the app using validated measures, and exploring user perspectives on how to make further improvements to the app.

**Methods:**

Caregivers of children with special health care needs were recruited to participate in a simulation exercise using Caremap to coordinate care for a simulated case of a child with complex medical and behavioral needs. Participants completed a postsimulation questionnaire adapted from 2 validated surveys: the Pediatric Integrated Care Survey (PICS) and the user version of the Mobile Application Rating Scale (uMARS). A key informant interview was also conducted with a liaison to Spanish-speaking families regarding app accessibility for non–English-speaking users.

**Results:**

A Caremap simulation was successfully developed in partnership with families of children with special health care needs. Overall, 38 families recruited from 19 different US states participated in the simulation exercise and completed the survey. The average rating for the survey adapted from the PICS was 4.1 (SD 0.82) out of 5, and the average rating for the adapted uMARS survey was 4 (SD 0.83) out of 5. The highest-rated app feature was the ability to track progress toward short-term, patient- and family-defined care goals.

**Conclusions:**

Internet-based simulation successfully facilitated end-user engagement and feedback for a digital health care coordination app for families of children with special health care needs. The families who completed simulation with Caremap rated it highly across several domains related to care coordination. The simulation study results elucidated key areas for improvement that translated into actionable next steps in app development.

## Introduction

### Background

Care for >13 million children with special health care needs in the United States [1] often requires their parents or caregivers to manage information and communication across a complex ecosystem of providers and disciplines located both within and outside of traditional health care settings. These include, but are not limited to, medical, social, behavioral, developmental, educational, home care, and supportive therapy providers [2]. The organization of such a complex care team requires effective care coordination, defined as a “patient- and family-centered, assessment-driven, team-based activity designed to meet the needs of children and youth while enhancing the caregiving capabilities of families” [2-5]. Effective care coordination is associated with better outcomes [3,4]; however, effective care coordination for children with special health care needs is challenging to implement and sustain at a systematic level.

### Promising Tools for Care Coordination for Families of Children With Special Health Care Needs

**Figure 1:**
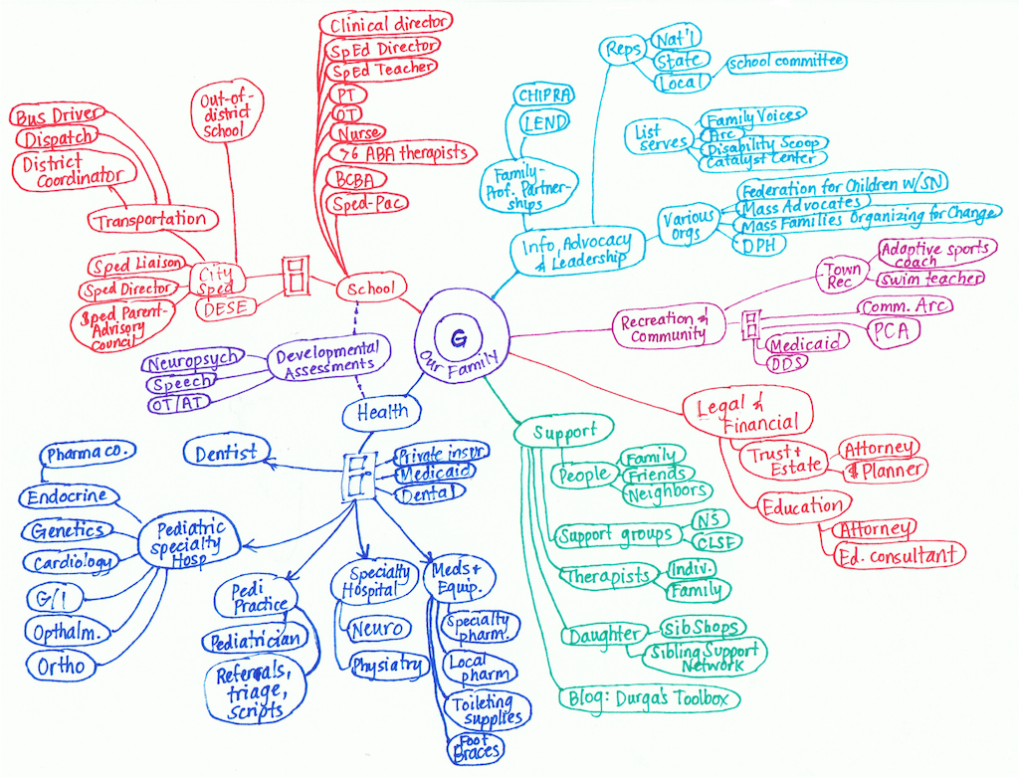
An example of a hand-drawn care map showing a network of support that contains various entities in health care, legal, financial, education, community, and other resources centered around the patient’s family (reproduced from Lind [8] with permission from Cristin Lind).

Two existing family-centered tools hold promise to facilitate effective care coordination for families of children with special health care needs: care binders and care maps. Care binders are manually organized by many families of children with special health care needs to manage their child’s care and organize relevant health information (history, procedures, and medications) over time and across different health care systems and providers [6,7]. Care mapping is a family-driven process that visually highlights a family’s needs, strengths, and interactions among all resources available to support the child (Figure 1 [8]) [3,9,10]. Field experience with patients and families has demonstrated that the process of manually creating a hand- or computer-drawn care map can help families of children with special health care needs to prioritize needs, identify service gaps, and facilitate communication with care teams to clarify roles, responsibilities, and desired outcomes [11]. Care mapping offers a summative overview of interrelated connections across each child’s multidisciplinary care team members. Coupled with the traditional, family-curated *3-ring binder*, care mapping offers the ability for a family to comprehensively archive health information and family-reported insights over time.

Although care binders and care mapping can play an important role in coordinating care for children with special health care needs, responsibility falls on families of children with special health care needs to create and maintain these tools for the organization of information across a complex network of services and providers. Furthermore, these tools are not integrated with electronic health records (EHRs). As a result, rich insights from caregivers contained within binders and care maps are difficult to share and largely invisible to health care providers across sectors, ultimately contributing to care fragmentation. Preserving the core benefits of these care coordination tools while concurrently identifying ways to scale up their use by both caregivers and health providers will be important to broaden access to family-driven and family-centered care coordination tools that may help to improve health outcomes for children with special health care needs.

Digital health tools developed to meet the care coordination needs of families of children with special health care needs [12,13] have the potential to support effective care coordination, but there has been limited research to date into the impact of these tools on outcomes. Currently, there is a dearth of measurable experiences of the implementation of digital health tools for children with special health care needs and their families [14]. Furthermore, despite growth in digital health tool development, there exist two major obstacles to broader real-world implementation of digital care coordination tools for children with special health care needs: (1) the lack of integration of patient- and family-reported content with EHR data and (2) the inability of health care providers to visualize insights recorded by patients and families over time.

### Caremap: A Digital Care Mapping and Health Binder Tool

A digital personal health record (PHR) is a digital health tool designed to securely access, manage, and share health information. In contrast to paper-based tools such as care mapping and care binders, a digital PHR can (1) access information across multiple health systems, (2) store and share family-reported health insights (eg, care goals and symptoms), and (3) facilitate communication with providers. A digital PHR can leverage Fast Healthcare Interoperability Resources (FHIR) open-data standards to securely access and share data among multiple EHRs and mobile apps [15]. FHIR standards are increasingly being adopted by health systems for digital health apps [16,17], and the 21st Century Cures Act now requires electronic health information to be shared freely with patients by health provider entities [18]. By applying these features to integrate the core benefits of care maps and care binders, digital PHRs are well positioned to facilitate care coordination for children with special health care needs [19].

Caremap [20] is an FHIR-enabled digital PHR mobile app designed by patients and families in collaboration with digital health developers, clinicians, and researchers to support family-centered care coordination for children with special health care needs and make family-reported longitudinal insights visible to health care providers. After initial development of the Caremap app, it was entered into the US Health Resources and Services Administration Maternal and Child Health Bureau’s Care Coordination for Children with Special Health Care Needs Challenge in 2019, where it was declared the winner by a panel of federal judges and expert advisors who evaluated the app’s potential for impact, sustainability, accessibility, and innovation in care coordination [13]. Before further testing in real-world clinical environments, rigorous proof-of-concept testing with families of children with special health care needs was identified as a critical next step.

User-centered design with early end-user engagement is increasingly recognized as essential for digital health tool development [21-24]. The goal of this study was to co-design with end users a simulation exercise to conduct proof-of-concept testing of the Caremap mobile app to coordinate care for children with special health care needs. The specific aims of the study included (1) engaging end users (ie, families of children with special health care needs) in app co-design using a mobile app simulation, (2) evaluating the usability of the app using validated measures of app usability and care integration, and (3) exploring user perspectives on how to make further improvements to the app using survey feedback and key informant interviews.

## Methods

### Setting and Approach

This was a prospective study with adults from families with children with special health care needs who participated in an internet-based simulation exercise to test the usability of the Caremap mobile app as part of the Maternal and Child Health Bureau’s Care Coordination for Children with Special Health Care Needs Challenge that sought innovative technology-based solutions to address care coordination for children with special health care needs [13]. Usability testing was originally intended to be conducted in person using real EHR data; however, because of the COVID-19 pandemic and the closure of many ambulatory clinics across the United States, the original in-person usability testing plan was adapted into an internet-based simulation exercise based on a simulated patient case.

### Rationale for Simulation as an Approach to End-User Testing

We selected simulation as the approach for usability testing because of its supporting evidence base as a methodology for user testing [25,26] and for the flexibility offered by simulation during the COVID-19 pandemic. Simulation offered a rapid and inexpensive technique to efficiently gather feedback from a broad sample of users from multiple regions, identify pain points, and make critical adjustments early in the app development process.

### Caremap Mobile App Description

**Figure 2:**
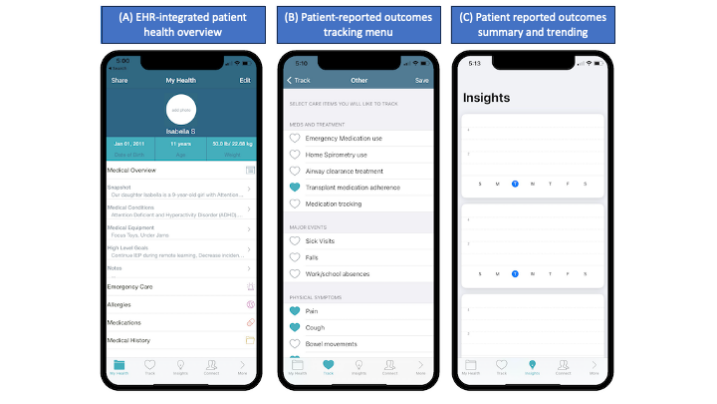
Select screenshots of the Caremap digital personal health record (PHR) mobile app. (A) My Health tab showing PHR features. (B) Track tab showing a sample of trackable family-reported health outcomes for users. (C) Insights tab showing a graphical summary design of user-tracked outcomes. EHR: electronic health record.

**Figure 3:**
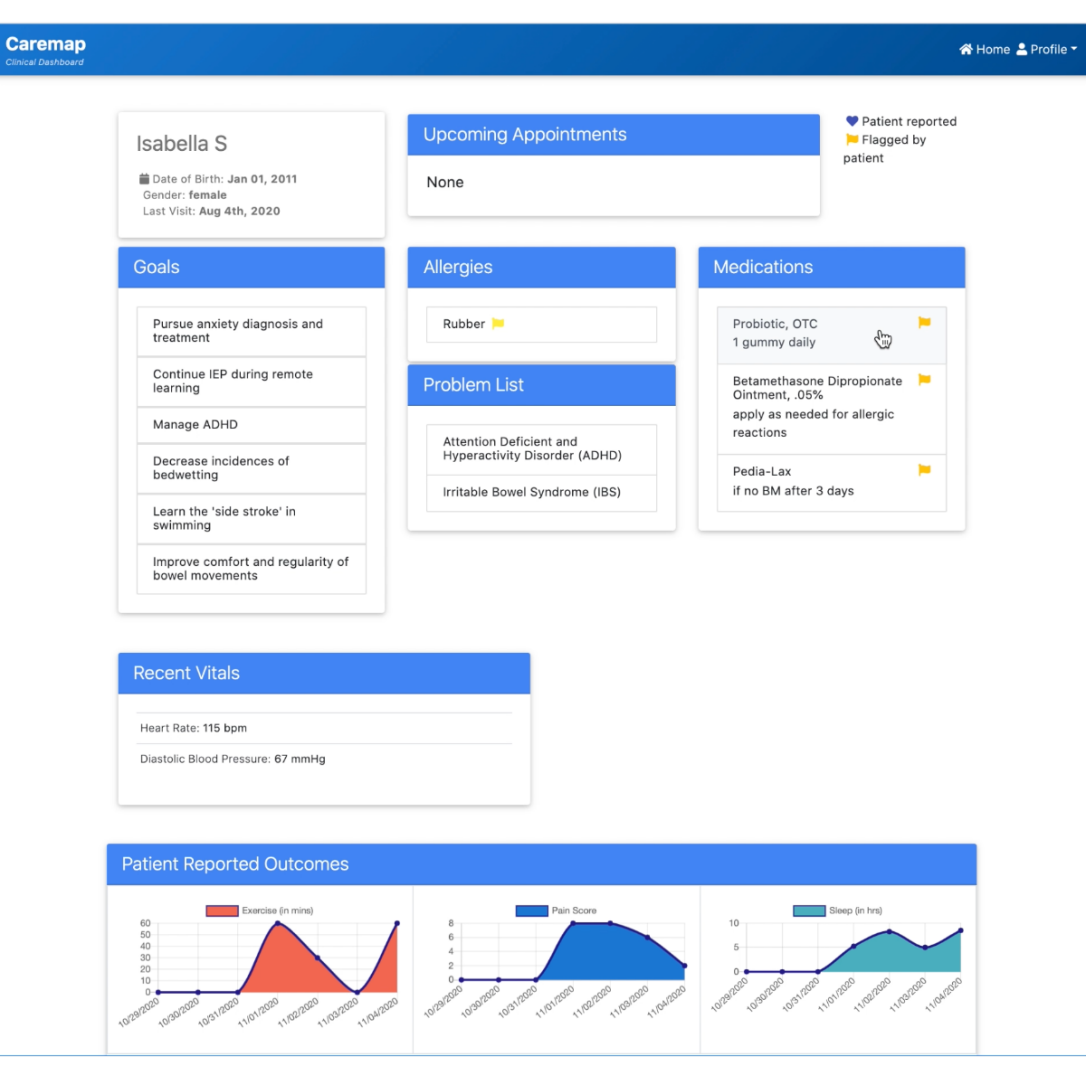
Interface of the Caremap prototype clinical dashboard to be integrated into an electronic health record showing an overview of the patient’s pertinent health information, including active care goals, allergies, problems list, and medications. The dashboard also shows items flagged by the Caremap mobile app user (yellow flags) for review. In addition, the dashboard summarizes family-reported outcomes for the patient.

Caremap is an iOS-compatible, FHIR-enabled mobile app that allows users to view and share a summary of their child’s latest health record, set patient- and family-centered care goals, track symptoms, monitor progress toward care goals, and share health data across care entities to ultimately facilitate care coordination across multiple care team members. Caremap includes 2 primary components: a patient-facing mobile app (Figure 2) and a clinical dashboard (Figure 3); only the patient-facing mobile app was assessed in this study. A detailed description of the core components of the patient-facing mobile app can be found in Multimedia Appendix 1.

### Study Participants and Recruitment

Participants in the simulation exercise for app testing were recruited using convenience sampling via direct email outreach to a national sample of families of children with special health care needs engaged with Family Voices. Family Voices [27] is a national family-led organization of friends and families representing, and providing support to, families of children with special health care needs. Family Voices leads a network of state-level organizations that are present in all 50 US states, 5 US territories, and 3 tribal communities. The inclusion criteria for participants in this study were being a family member caring for a child or youth with special health care needs, having access to the requisite technology (iOS-compatible device and internet access), and being able to speak English. Upon the completion of the simulation exercise, participants received compensation consistent with best practice standards for family-engaged research [28]. In total, 38 families were recruited into the study. Individual participant-level demographics were not collected to respect the privacy of the families recruited.

### Simulation Exercise Design and Implementation

The testing version of Caremap included a prepopulated complete record of information about a simulated pediatric patient with complex behavioral and medical needs, including information about medical conditions, medical equipment, patient goals, emergency care, allergies, medications, hospitalizations, and the care team. The cocreation of the simulated patient case and fictitious biopsychosocial elements involved an interdisciplinary team of families of children with special health care needs from Family Voices and the family advisory committee of Boston Children’s Hospital, clinicians and researchers from Boston Children’s Hospital, and an elementary special education teacher. The simulated patient case was reflective of the lived experience of, and real-world challenges faced by, children with special health care needs and their families.

Between October 12, 2020, and November 2, 2020, the participating families downloaded and used the Caremap mobile app to complete the simulation exercise. For the simulation, participants assumed the role of a family to the simulated child with complex needs, *Isabella* (aged 9 years), and used the Caremap mobile app to review the case details on their personal devices. Participants were invited to familiarize themselves with the Caremap app on their own, after which they were provided with specific activities within Caremap to coordinate *Isabella’s* care in response to simulated real-world scenarios that demonstrate use cases for Caremap’s mobile features. These activities included (1) reviewing *Isabella’s* profile and making additions and changes to health data such as medications and medical history; (2) tracking medication use and symptoms related to *Isabella’s* health, including urination frequency, mood, bowel movements, and sleep over the last 3 days; (3) reviewing simulated data graphics based on previous simulated data entries; and (4) communicating and sharing health data with other care team members via the app. The simulation was designed to take around 30 minutes. Immediately after the simulation experience with the Caremap app, participants were invited to complete a 29-item web-based survey to gather family-reported feedback. Complete simulation instructions for participants can be found in Multimedia Appendix 2.

### Outcomes or Measures

Study data were collected and recorded on the web using REDCap (Research Electronic Data Capture; Vanderbilt University) [29]. The survey had two parts: (1) an *experience of care integration* instrument containing 12 questions adapted from the Pediatric Integrated Care Survey (PICS), a validated instrument that captures family-reported experiences with care integration (ie, working with their child’s care team to plan, manage, and track their child’s care) [30], and (2) a *functionality assessment* instrument containing 17 questions adapted from the user version of the Mobile Application Rating Scale (uMARS) [31], a validated tool that assesses mobile app usability. The final study survey items (Multimedia Appendix 3) were adapted by the research team, which included investigators and family partners, and vetted with Family Voices; for example, the PICS question “In the past 12 months, how often have you felt that your child’s care team members thought about the ‘big picture’ when caring for your child, meaning dealing with all of your child’s needs?” was adapted in this study to “Based on your experience using Caremap, how easy or difficult would it be for Isabella’s family to make sure that the ‘big picture’ was taken into account when decisions and recommendations were made about Isabella’s care?” The adapted survey questions were a combination of 5-point Likert scale questions and sliding scale questions rated from 0 to 100. The questions were also grouped into the following themes from the original uMARS instrument: engagement, functionality, aesthetics, information, and subjective quality, as well as perceived impact (not part of the uMARS).

In addition, participants responded to 3 open-ended questions associated with the *functionality assessment* instrument on app functionality to evaluate their perceived level of understanding of the opportunities in care coordination after the simulation, their confidence in the app’s ability to coordinate care across team members, and their confidence in their own ability to coordinate care. Refer to the full survey in Multimedia Appendix 3 for more details.

### Key Informant Interview

In addition to the user-reported measures of app usability and care integration, a key informant interview was conducted with a liaison to Spanish-speaking families of children with special health care needs at Boston Children’s Hospital to assess the potential benefits and challenges of Caremap regarding advancing health equity, particularly for Spanish-speaking families of children with special health care needs.

### Analysis

Quantitative data collected from the adapted PICS and uMARS survey were summarized using descriptive statistics. Sliding scale responses were converted to 5-point Likert scores for ease of comparison (eg, scores of 0-20 on the sliding scale=1 out of 5 on the Likert scale). Top and bottom box analysis of the adapted PICS and uMARS survey results was also conducted, where the top box responses represented the 2 most favorable responses for each question (ie, Likert score of 4 or 5) whereas the bottom box responses represented the 2 least favorable responses (ie, Likert score of 1 or 2) [32]. Free-response answers from the *functionality assessment* survey were categorized by *constructive* versus *positive* commentary, and thematic analysis was conducted on these responses by 1 reviewer for key themes and verified by 2 other reviewers independently. Insights from the key informant interview were summarized into key learnings and translated directly into actionable steps for app improvements.

### Ethical Considerations

The study was reviewed by the Performance Excellence Group at Boston Children’s Hospital, and it was determined to be exempt from institutional review board review.

### App Security Assessment, Informed Consent, and Participation

Caremap was reviewed with the Boston Children’s Hospital information security team. The review included a thorough assessment of the overall solution, validation of the data security protections in place, identification of the use of protected health information in the solution by patients and care teams, and vulnerability code scanning for the app. The review resulted in the approval of the app for the limited scope of the simulation study. Protected health information was not used in the simulation, and participants did not have the ability to upload or import personal information into the app during simulation testing. Participants were required to read through, and acknowledge, a consent letter describing the risks and benefits of the simulation study before participating in it. Parent and caregiver participants received US $100 in compensation after the completion of the simulation exercise and all postsimulation surveys.

## Results

**Figure 4:**
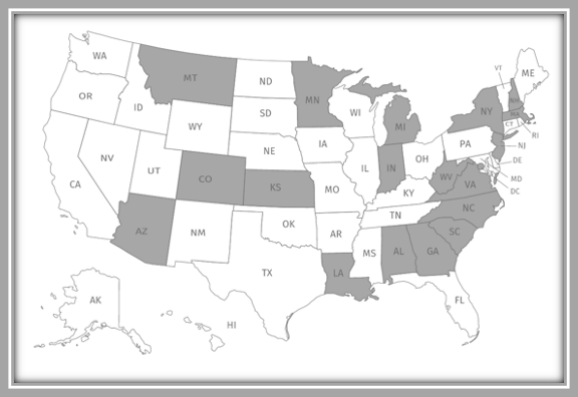
Map of participants recruited by US states highlighted in gray.

Over 1 month, from October 13, 2020, to November 12, 2020, a total of 45 families signed up for the simulation, and 38 (84%) families from 19 states (Figure 4) completed both the simulation exercise and the survey. On the basis of aggregate internet connectivity data by state, participants spent an average of 34 minutes reviewing the simulated patient’s health information and using the Caremap app during the simulation.

### User-Reported Experience of Care Integration and App Functionality

The survey results from the participants (N=38) based on the *experience of care integration* and *functionality assessment* instruments are summarized in Tables 1 and 2. The responses from the *experience of care integration* survey demonstrated an average score of 4.1 (SD 0.82; range 3.8-4.5) out of 5 across all measures (5=very easy and 1=very difficult). Top and bottom box analysis revealed that the most highly rated capabilities included the app’s ability to track progress toward short-term goals (question 9, with a rating of 95% of the responses in the top box) and the ability for families to communicate with care teams regarding concerns about care (question 1, with a rating of 84% of the responses in the top box). The lowest-rated capabilities included the app’s ability to help families think about future care needs to ensure that they are taken care of (question 11, with a rating of 11% of the responses in the bottom box) and the ability to make sure that the *big picture* was considered when decisions were made for care (question 12, with a rating of 11% of the responses in the bottom box).

The *functionality assessment* survey showed an average app usability rating of 4 (SD 0.83; range 1-5) out of 5, with the perceived impact of the app being the highest-scoring category. Participants also rated the app highest in areas of engagement (out of 5: mean 4.1, SD 0.82; range 2-5), perceived impact (out of 5: mean 4.3, SD 1.18; range 2-5), and information quality (out of 5: mean 4.2, SD 0.70; range 1-5). Participants rated the app lowest in terms of functionality (out of 5: mean 3.8, SD 0.76; range 2-5) and aesthetics (out of 5: mean 3.9, SD 0.81; range 2-5).

**Table 1:** Summary of the experience of care integration survey (adapted Pediatric Integrated Care Survey [PICS]).

Question	PICS domain^a^	Category score (Likert scale), mean (SD)	Likert score, mean (SD; range)	Rating in the top 2 box (%)	Rating in bottom 2 box (%)
1	Care team communication	4.2 (0.82)	4.3 (0.85; 2-5)	84	5
2	Care team communication	4.2 (0.82)	4.2 (0.77; 2-5)	79	3
3	Care team communication	4.2 (0.82)	4.1 (0.74; 2-5)	82	3
4	Care team communication	4.2 (0.82)	4.1 (0.91; 2-5)	71	5
5	Team functioning, performance, quality, and connectivity	4.1 (0.85)	4.0 (0.82; 2-5)	74	5
6	Family impact	3.9 (0.81)	4.0 (0.81; 2-5)	79	5
7	Family impact	3.9 (0.81)	3.9 (0.81; 2-5)	66	3
8	Team functioning, performance, quality, and connectivity	4.1 (0.85)	4.1 (0.87; 2-5)	74	5
9	Care goals and care planning	4.3 (0.72)	4.5 (0.60; 3-5)	95	0
10	Care goals and care planning	4.3 (0.72)	4.1 (0.82; 2-5)	68	3
11	Family impact	3.9 (0.81)	3.8 (0.81; 2-5)	76	11
12	Integrator	3.8 (1.01)	3.8 (1.01; 1-5)	68	11

^a^Domains based on the PICS developed by Antonelli et al [32].

**Table 2:** Summary of the functionality assessment survey (adapted user version of the Mobile Application Rating Scale [uMARS]).

Question	uMARS domain^a^	Category score (Likert scale), mean (SD)	Likert score, mean (SD; range)	Rating in top 2 box (%)	Rating in bottom 2 box (%)
1	Engagement	4.1 (0.82)	4.4 (0.82; 2-5)	89	6
2	Engagement	4.1 (0.82)	3.8 (0.90; 2-5)	69	11
3	Engagement	4.1 (0.82)	4.3 (0.72; 2-5)	89	3
4	Functionality	3.8 (0.76)	3.7 (0.79; 2-5)	71	8
5	Functionality	3.8 (0.76)	3.9 (0.73; 2-5)	63	3
6	Aesthetics	3.9 (0.81)	4.0 (0.91; 2-5)	63	3
7	Aesthetics	3.9 (0.81)	3.9 (0.69; 3-5)	71	0
8	Information	4.2 (0.70)	3.9 (0.80; 1-5)	82	6
9	Information	4.2 (0.70)	4.2 (0.69; 3-5)	84	0
10	Information	4.2 (0.70)	4.5 (0.61; 3-5)	84	0
11	App subjective quality	3.9 (1.05)	4.3 (0.86; 2-5)	79	3
12	App subjective quality	3.9 (1.05)	4.0 (0.96; 2-5)	66	6
13	App subjective quality	3.9 (1.05)	3.3 (1.27; 1-5)^b^	26	13
14	Perceived impact	4.2 (1.10)	4.2 (1.09; 1-5)^b^	63	5
15	Perceived impact	4.2 (1.10)	4.2 (1.10; 1-5)^b^	58	6
16	Perceived impact	4.2 (1.10)	4.3 (1.18; 1-5)^b^	68	6
17	Perceived impact	4.2 (1.10)	4.2 (1.02; 2-5)^b^	61	0

^a^Domains based on the user version of the uMARS [31].

^b^Likert scores converted from a sliding scale score based on the process outlined in the *Methods* section.

### Thematic Analysis of Free-Response Commentary by Simulation Participants

The identified core themes from the thematic analysis along with representative participant comments are shown in Textbox 1. Positive feedback highlighted that Caremap (1) would make coordinating care easier for families of children with special health care needs, (2) is a convenient way to store and manage health care data, (3) facilitates improved communication with care teams, and (4) promotes shared understanding of care plans that could prevent inadvertent misses by providers and enhance care safety. The positive commentary repeatedly pointed to Caremap’s advantages in creating (1) accessibility for parents with busy schedules, (2) central information storage capability, and (3) capability to share knowledge with both providers and other caregivers, facilitating cross-stakeholder understanding. The constructive feedback indicated a need for integration of the Caremap mobile app with existing secure messaging platforms (eg, patient portals) to streamline communication with providers; participants also provided recommendations for functionality improvements that would enhance the app user experience (UX; eg, direct import of contacts within the device used). Additional constructive feedback highlighted the importance of integrating Caremap into the existing life structure of families of children with special health care needs without creating added burden as well as customizability of app features and UX to fit specific end users’ needs.

**Results of thematic analysis and representative commentary.**

- Caremap mobile app makes coordinating care easier for families of children with special health care needs
  - “This app would make organizing and coordinating my child’s health care needs so much more effective, streamlined and all around easier. As parents if [of] special needs kiddos, our brains are always so full of information...info we need to tell doctors, info we need to tell therapists, Info [info] we think of we [at] 3am and have to then try to remember to tell ours [our] kiddos team are [at] a more reasonable hour in the day...if we had this app, we could click in at 3am and get all of those things off our mind, and the info would be there for the providers to see! Life changing!!”
- Caremap mobile app is a convenient way to store and manage health care data
  - “Absolutely!! Everything in one user friendly app. It would help when my husband had to take our child to appointments too. He could always be in the loop with what is going on. And have access to the information if he didn’t know an answer to a question.”
- Caremap mobile app facilitates improved communication with care teams
  - “Often times health professionals do not review charts in advance or don’t understand the bigger picture and the goals we have. This would be an excellent way to share those and encourage parents to speak up without feeling uncomfortable doing so.”
- Caremap mobile app promotes shared understanding of care plans to prevent inadvertent misses and enhance safety
  - “Coordinating with all of the specialists, Teachers, therapists, etc is one of the most time consuming and frustrating tasks of a special needs parent. Streamlining coordination would safe [save] so much time and prevent so many misunderstandings and inadvertent ‘misses’ in the health care plan.”
- Need for integration of Caremap into existing tools for health care coordination without added burden on end users
  - “I feel like this is a proof of concept for an app that ultimately could be integrated into something like a secure messaging platform for communicating within the app, allowing all care info to be centralized. I’d like to see something like a ‘visibility’ radio [button] selection for data in the app that would automatically share it to different family/care team members (either using the same app or a compatibly [compatible] platform in a medical facility). As is, it seems like it’s a good activity tracker combined with a limited note taking app and an address book.”
- Need for customizability of app user interface and functionality to match end-user needs
  - “Thoughts on provider info and communicating with providers: Would be great to import provider contacts directly from phone contacts. Also would be helpful to put in next appt [appointment] with providers. Relationship field: leave a blank option to fill in. It’s impt [important] to know the provider specialty, but there’s too many for a drop down menu. Provider section also needs a blank notes field re: office, parking, nurse info. I find myself strongly preferring to contact our 20+ providers via online portal rather than phone. The feasibility of integrating this app with secure portals is likely a challenge. This limitation would limit my use of the app as a primary communication tool.”
  - “Ideas for improving: Add height to opening dashboard, as height and weight are both regularly required for monthly supply orders. Calendar integration would be great. Tracking appts [appointments] is the main reason I keep an online calendar. Would like an option to mark issues/goals/meds [medications] and export those into a checklist to guide an individual provider appt [appointment]. For discharge instructions, I would like to see a photo option, since those are often long and come in paper form.”

### Key Informant Interview

Learnings from the key informant interview with a liaison to Spanish-speaking families of children with special health care needs at Boston Children’s Hospital are summarized in Textbox 2. These key themes were directly translated into actionable next steps for development in partnership with our key informant to enhance equitable access to Caremap.

**Key informant interview learnings and next steps.**

- Solution must be accessible to users with limited tech literacy and a tool that supports, not burdens, families
  - Need user support system onboarding new families; design must be simple to navigate (eg, yes or no answers)
- Platform needs to address cultural uniqueness and the ways families write, read, and absorb information
  - Build out “About Me” field within Caremap app to account for cultural preferences and attributes
- Health education and information must be presented at comprehension levels that facilitate understanding
  - Continue to translate and refine Caremap language and prompts to facilitate easy user experience for all families
- Access to child’s medical information (eg, medications and care team member details) would facilitate improved family-provider discussions, especially in emergency department and urgent settings
  - Must include accessible information that matters most to all families in clinical situations; ensure broad access by making app available on lower-cost devices

## Discussion

### Overview

This study used simulation to gather formative feedback from a national sample of caregivers of children with special health care needs on the usability and functionality of a digital PHR mobile app, Caremap. The findings reported by users during testing provided support for the proof of concept of the Caremap mobile app as a care coordination tool for children with special health care needs. Our use of simulation as a method for user engagement in the mobile app design process and our description of this novel approach can help inform other research teams leading mobile app development.

### Principal Findings

Early end-user engagement in health innovations and technology development is highly important [21-23], yet there is limited literature describing end-user contributions to digital health tools for children with special health care needs [14]. This study was innovative because it demonstrated the feasibility of leveraging an internet-based app simulation co-designed with families of children with special health care needs as a user-centered approach to gather early formative feedback on the usability of a digital care coordination tool for children with special health care needs. Furthermore, this study demonstrated the effectiveness and flexibility provided by a simulation-based research method over traditional recruitment methods to quickly recruit participants nationally (eg, successful recruitment of 38 families of children with special health care needs from 19 states within 6 weeks with a completion rate of 38/45, 84%). Partnership with a family-led community-based group (Family Voices) representative of our target end users was a key strategy that enhanced study recruitment. The success of this user-engaged recruitment strategy can serve as a framework for future studies evaluating digital health tools serving targeted clinical populations such as children with special health care needs.

Family-reported responses to survey items revealed high ratings from families of children with special health care needs across multiple domains. On the basis of the experience of the care integration survey (adapted PICS), families rated Caremap particularly highly in the *care goals/care planning* domain (out of 5: average 4.3, SD 0.72; range 4.1-4.5, across domain questions) as a tool to help track short-term care goals and as a highly engaging tool for care coordination. This was further reinforced as a strength of the app by the open-ended responses, with positive participant comments on the advantage of the tracking capability in Caremap, revealing how “daily health tracking [has become] a necessity” and that “there’s value in [Caremap]...as it would help me track progress/problems/etc. for appointments.” This demonstrates a real need among families of children with special health care needs for an improved tool to record care goals, symptoms, and medications in real time.

The adapted PICS responses and open-ended participant comments also revealed that Caremap is promising as a tool to enhance communication with care team members, with high ratings in the *care team communication* domain (out of 5: average 4.2, SD 0.82; range 4.1-4.3). The open-ended participant feedback highlighted the role that asynchronous access to editing patient health data can play in communication with providers; as a participant noted, “if we had this app, we could click in at 3am and get all of those things off our mind, and the info would be there for the providers to see! Life changing!!” Other commentary highlighted ways in which even in-person communication could be enhanced by Caremap, with a participant stating that “often times health professionals...don’t understand the bigger picture and the goals we have. [Caremap] would be an excellent way to share those and encourage parents to speak up without feeling uncomfortable doing so.”

On the basis of responses to the *functionality assessment* survey questions, the app scored highly in the *information* domain (out of 5: average 4.2, SD 0.70; range 3.9-4.5, across domain questions). Participants rated the app particularly highly as a credible source of information (question 10, out of 5: average 4.5, SD 0.61; range 3-5). This is an exciting early finding, indicating that Caremap and other digital health tools may be a viable way for health professionals to deliver information quickly and efficiently to families of children with special health care needs. In addition, the participants rated the app highly in the *perceived impact* domain (out of 5: average 4.2, SD 1.10; range 4.2-4.3, across domain questions). However, the SDs on these questions were high (1.09-1.18), indicating some discrepancy among participants on the app’s future care coordination value. Given the aforementioned evidence of Caremap’s tracking feature as a core advantage, this may indicate that Caremap can better serve certain populations of children with special health care needs where tracking of symptoms, medications, and goals is of particular importance (eg, children with cystic fibrosis needing to track pulmonary function and bowel movements to tailor medical management).

Participants rated the app highly in the *engagement* domain (out of 5: average 4.1, SD 0.82; range 3.8-4.4, across domain questions), particularly when asked whether Caremap is an interesting app to use (functionality assessment survey, question 1, out of 5: average 4.4, SD 0.82; range 2-5). Although the simulation was designed to last 30 minutes, it was affirming to see that some participants actively used the app for simulation for >40 minutes. However, these findings are based on a time-limited experience of a simulation exercise and prone to social desirability bias. Long-term app engagement, a common pitfall among digital health tools [33], could not be measured. This will need to be further evaluated with future studies.

Importantly, feedback from the users in our simulation study elucidated key areas for development; for example, the app received a low rating in the adapted PICS (Table 1) *integrator* domain (out of 5: average 3.8, SD 1.01; range 1-5) for the ability to help care teams keep the *big picture* in mind for care decision-making, as well as low scores in the *family impact* domain (out of 5: average score 3.9, SD 0.81; range 3.8-4.0) regarding communicating with care team members on high-level issues such as challenges at home for the family in caring for their child. This could suggest that future versions of Caremap need to place a family-written narrative that summarizes key care goals and priorities for the child front and center for the care provider to view. In addition, these low scores may be related to concerns from participants regarding Caremap’s ability to integrate with existing care coordination tools such as MyChart, limiting its use to engagement with care team members. This was compounded with expressed concerns from participants regarding provider engagement, as evidenced by comments such as “for full functionality, [parents] need buy in from the care providers” and “I hesitate it will be easy to contact providers [because] when you call, email or use this app, it can be difficult to get ahold of sometimes and listen and follow through on concerns.” This highlights the need for further trials with health providers using Caremap for care coordination to understand the facilitators and barriers to provider engagement, a key component of digital health tools to improve care coordination [19].

In addition, in the *functionality assessment* survey, participants generally rated the app less favorably in functionality (questions 4 and 5) and aesthetics (questions 6 and 7). Particularly low scores on questions 4 and 5 suggest that participants found it difficult to navigate the app’s user interface (UI). The constructive comments by participants included a recommendation for a UI or UX enhancement; for example, a participant expressed the desire for the ability to toggle visibility between shared and unshared data. In addition, several participants commented about experiencing crashes and bugs during the simulation. This feedback suggests that UI and UX development are top of mind for participants, and further development to ensure logical UI and UX and minimal bugs is needed before a full-scale launch. Prioritizing UI and UX will also be highly important moving forward because users are more likely to engage with mobile health apps that match user expectations in terms of app looks and functionality based on popular industry-developed apps [34]. Insights from our in-depth interview with the hospital liaison for Spanish-speaking families also highlighted how multicultural and socioeconomic accessibility needs to be a priority moving forward, including building in languages other than English and compatibility with non-iOS devices. This feedback is in line with current studies that suggest that mobile apps are not usable for diverse populations as they are designed currently [35]. This emphasizes the need to engage with a more diverse user population in future app co-design and testing to generate broader insights on the barriers to use for non–English-speaking families and families with low-income status, as well as other diverse populations.

### Strengths and Limitations

This study has several strengths. First, our user-engaged approach in partnership with Family Voices, a national advocacy group for families of children with special health care needs, allowed for cocreation of a comprehensive and representative patient case for the simulation exercise and facilitated the nationwide recruitment of participants who had lived experience as caregivers for children with special health care needs. Another strength of this study was the use of validated measures to evaluate the participant experience of care integration and mobile app usability with questions adapted from the PICS and uMARS, respectively, improving the app’s comparability with other digital health tools. Finally, the use of a digital simulation was a novel method for user engagement that facilitated flexibility and efficiency in study recruitment during the COVID-19 pandemic.

Several limitations of this study should also be considered. First, there was a risk of social desirability bias in terms of participants providing positive feedback because they may have perceived a high degree of investment in developing this app by the study team. To mitigate this risk, participants completed the simulation exercise and survey from home without contact from the study team or direct interaction with other study participants to ensure that the responses more likely reflected their own thoughts. In addition, the simulation exercise did not include actual use and testing of FHIR-enabled EHR integration, and issues surrounding integration in real-world implementation could not be elucidated. Furthermore, the Caremap app was only available in English and required an active internet connection at home for testing, which excluded the participation of non-English speakers and individuals without personal devices or internet connectivity. In addition, clinicians’ perceptions of usability were not included in this study, but gathering clinician perspectives will be a key future direction for continued app development. Finally, owing to the time-limited nature of the simulation exercise, long-term user engagement and impact have yet to be elucidated regarding Caremap as a tool for care coordination. However, this study was designed as a proof-of-concept exercise to serve as a foundation for future longitudinal studies that will further elucidate this aspect.

### Next Steps

Future studies will need to be conducted to evaluate real-world implementation using structured implementation (eg, Consolidated Framework for Implementation Research) and digital health adoption (eg, Technology Acceptance Model) frameworks to understand the elements of patient and provider engagement with Caremap for care coordination. Future testing will need to include real-world testing of FHIR-enabled integration to validate end-to-end connectivity between Caremap users and care providers. This will include conducting prospective trials with health providers who serve children with special health care needs to test the embedded clinician dashboard. Prospective testing will also need to measure the impacts of the Caremap platform on meaningful patient- and family-centered outcomes and include diverse users reflective of the population of families of children with special health care needs. In 2021, the National Quality Forum, with the support of the Centers for Medicare & Medicaid Services, convened a multistakeholder group of patients, advocates, informaticians, and multidisciplinary care providers and subsequently released a set of recommendations on how EHRs can be used to facilitate improved care communications and care coordination, including (1) collecting and sharing standardized data, (2) optimizing EHR usability for patients and caregivers (3) optimizing EHR usability for clinicians, (4) developing novel EHR data elements to improve measurement, and (5) leveraging EHR data to fill measurement gaps [19]. Future studies will need to elucidate the ways in which Caremap can be developed to best align with these recommendations [19].

This proof-of-concept simulation study has also helped to uncover specific areas for future investigation. Future work will need to include the exploration of improvements to the in-app UX through enhanced app aesthetics; family-centered communication with care providers; and additional app functions such as goal sharing, task delegation, calendar integration, and contingency plan documentation. Enhancing equitable access to this app will be a priority that will be addressed in future development, such as adapting the app for the Android platform, updating in-app language to enhance use for those with limited health literacy, and creating multilingual options (eg, a Spanish version). In future studies, demographic information of study participants should also be collected to examine differences in app engagement among various patient and caregiver subpopulations. As provider engagement will also be key to care coordination through Caremap, future studies will need to include health providers to uncover barriers and facilitators to their app engagement and use.

### Conclusions

In this paper, we describe the use of simulation to gather end-user feedback on the usability and functionality of Caremap, a novel mobile app designed by families, clinicians, and technology experts to improve care coordination for children with special health care needs. In this simulation exercise, a national sample of caregivers of children with special health care needs rated a novel FHIR-enabled mobile app highly as a usable tool for care coordination with strong potential to improve integrated care across sectors. This user-centered approach offered important insights into usability and proof-of-concept testing before the real-world implementation of the mobile app.

## Appendix

Multimedia Appendix 1 Detailed description of the Caremap mobile app.

Multimedia Appendix 2 Caremap simulation experience.

Multimedia Appendix 3 Adapted Pediatric Integrated Care Survey (PICS) and user version of the Mobile Application Rating Scale (uMARS) questionnaire (Caremap simulation feedback survey).

## Abbreviations

EHR: electronic health record
FHIR: Fast Healthcare Interoperability Resources
PHR: personal health record
PICS: Pediatric Integrated Care Survey
REDCap: Research Electronic Data Capture
UI: user interface
uMARS: user version of the Mobile Application Rating Scale
UX: user experience

## Data Availability

The data sets generated and analyzed during this study are available from the corresponding author on reasonable request.
