# Supplementary material for: Outcomes of End-User Testing of a Care Coordination Mobile App With Families of Children With Special Health Care Needs: Simulation Study
Source: JMIR Form Res. 2023 Aug 28;7:e43993. doi: 10.2196/43993 (PMC10495855; doi:10.2196/43993)
Supplement: Multimedia Appendix 1 [file formative_v7i1e43993_app1.pdf]

Multimedia Appendix: Caremap Mobile Application Detailed Description

Data Integration: Patient directed EHR data sharing is enabled by 1upHealth that leverages Fast Healthcare Interoperable Resources (FHIR) to support seamless exchanges of health data between patients and providers. Currently, over 200+ health systems are compatible with Caremap©.

Personal Health Record ('My Health' tab): Integrated data across multiple health providers are compiled into Caremap© as a singular, up to date record of the patient's health. This information includes a family-written patient summary and care goals, medical conditions, allergies, medications, recent admissions, and procedures (figure below, left). Caremap© also contains emergency care instructions for scenarios pertinent to the patient (e.g. peanut allergy anaphylactic shock protocol) that is sharable with others as a Portable Document Format (PDF) directly from the app. PHR data can be personalized by the user by flagging information for review with a clinician, record additional information (e.g. add new over the counter medications), or recording notes that can be shared with the care team.

Health Tracking Tool ('Tracking' and 'Insights tabs): Caremap© allows users to track select family-reported health measures, such as pain, nausea, missed school days, medication adherence etc. according to their care priorities and goals (figure below, middle). These outcomes are summarized in graphical form for the user (figure below, right) and viewable from the corresponding clinician's dashboard. Users are also able to set notification thresholds for reminders to contact or discuss with a health provider.

Care Team Communication ('Care team' tab): The Caremap© mobile app also contains a list of contacts pertinent to the care of their child for quick reference and direct communication that is integrated with iOS that is customizable by the user.

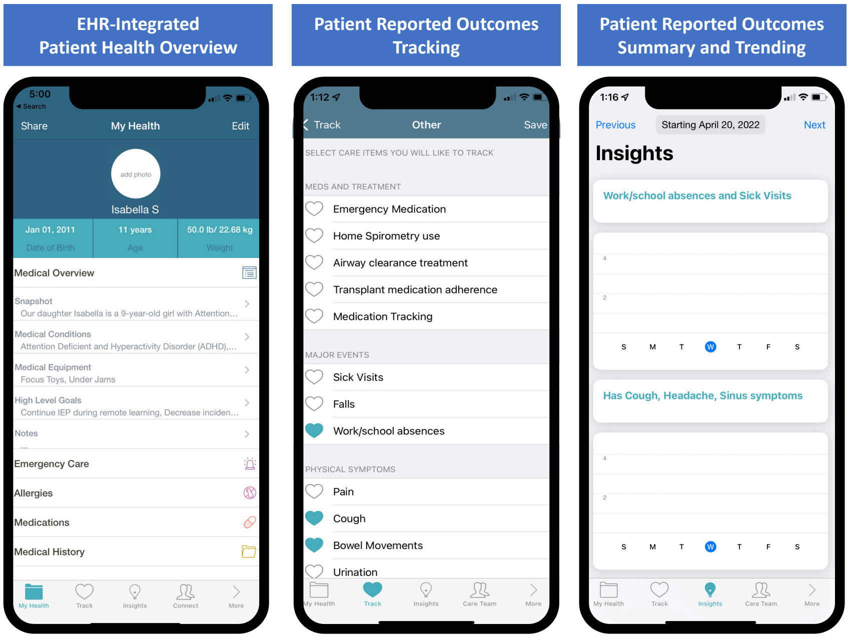

This is a Multimedia Appendix to a full manuscript published in the J Med Internet Res. For full copyright and citation information see http://dx.doi.org/10.2196/jmir.43933
