# Supplementary material for: Outcomes of End-User Testing of a Care Coordination Mobile App With Families of Children With Special Health Care Needs: Simulation Study
Source: JMIR Form Res. 2023 Aug 28;7:e43993. doi: 10.2196/43993 (PMC10495855; doi:10.2196/43993)
Supplement: Multimedia Appendix 2 [file formative_v7i1e43993_app2.pdf]

Caremap Simulation Experience

Welcome to the Caremap Simulation!

To participate, you will need:

- An iPhone or iPad
- These instructions either printed or loaded on another screen

This simulation is divided into five parts:

Part	Activity	Estimated Time
1	Downloading the Caremap App	5 minutes
2	Setting up the Simulation	5 minutes
3	Exploring the Caremap App	10 minutes
4	Using Caremap to Manage a Patient's Care	20 minutes
5	Completing the Feedback Survey	20 minutes

This exercise **does not** need to be completed all at once; you are welcome to move through the Simulation at your own pace and return to the instructions over time

Starting at Part 3, symbols are used to help guide you through the Simulation.

- ✓ Checkmarks indicate an action that you should complete in the Caremap App
- ➢ Arrows indicate tips for using the Caremap App (*Actions are optional*)
- ❖ Diamonds indicate additional information about the Caremap App (*No action is required*)

If you have a technical issue or question regarding the Simulation instructions, do not hesitate to reach out to the team at integratedcare@childrens.harvard.edu

Boston Children's Hospital
Until every child is well

FAMILY VOICES®

DukeHealth

PART 1: DOWNLOAD CAREMAP APP – 5 Minutes

Before beginning the Simulation Experience, you need to download the Caremap app on an Apple Device within a special testing application. If you experience issues opening the Caremap app, please delete the app from the iPhone or iPad and try reinstalling it. If you require further assistance, contact integratedcare@childrens.harvard.edu

1. Using Safari (Apple's default iOS web browser), open the following link: https://testflight.apple.com/join/BtW2vR0k

2. Under **'Step 1 Get TestFlight',** click **'View in App Store'.** Download **'TestFlight'** by clicking the cloud icon. Once downloaded, click **'OPEN'**, select the **'Don't Allow'** notifications option, and click **'Continue'**.

**If needed, use your own App Store username and password to install TestFlight.*

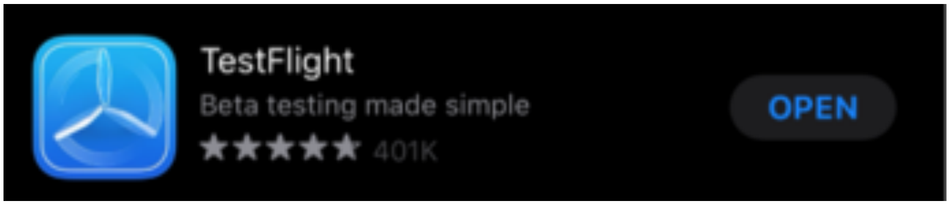

3. Exit **'TestFlight'** and return to the website: https://testflight.apple.com/join/BtW2vR0k

4. Proceed to **'Step 2 Join the Beta'** by clicking **'Start Testing'**. TestFlight will reopen automatically and the Caremap App will appear. Click 'ACCEPT', then 'INSTALL', and then 'OPEN'.

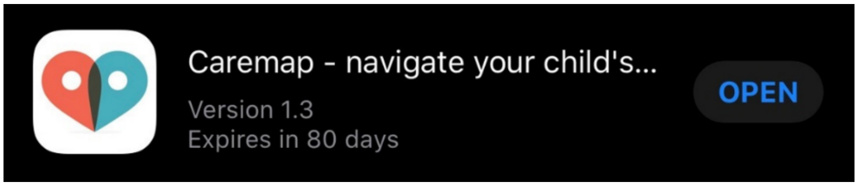

5. After selecting **'OPEN'**, a screen will pop-up with a note **'From the Developer'**. Click **'Next'** to continue and then click **'Start Testing'**.

6. Continue to **'CAREMAP SIMULATION SETUP'** for next steps.

PART 2: CAREMAP SIMULATION SETUP – 5 Minutes

Now that you have downloaded the Caremap app, please follow the instructions below.

1. Swipe left four times to view the **'intro pages'** that explain the Caremap app's functionality. When you get to the last page, click **'Sign In'**.

2. Sign into Caremap using our 'Simulation User Account'

Username: **sim-one@caremap.health**
Password: **Caremap2020**
(please note that the 'C' in 'Caremap2020' must be capitalized)

3. A **'User Profile'** page will appear. Click **'Next'.** Read the **'*Participation Agreement*'** and click the **'I acknowledge'** button in blue on the lower right hand side of the screen.

4. Read the overview below of your fictional child, Isabella S. After reading the overview, follow the instructions on the next page and proceed as if you are Isabella's Mom or Dad.

PATIENT OVERVIEW:

Isabella is a 9-year-old female with ADHD and possible Generalized Anxiety Disorder (GAD). She is incontinent at night requiring her to wear overnight protection and has periods of irritable bowel syndrome; both issues have been explored by urology and GI with negative findings. Her school psychologist and PCP suggest GAD may be the cause of these symptoms but Isabella's BCH Psychiatry appointment was cancelled upon the COVID-19 outbreak. She has a new virtual appointment on November 27, 2020.

Isabella is the oldest of 3 children; she has a 6-year-old sister and a 3-year-old brother. The children are in a 2 parent family, but after divorcing, mom and dad live in different houses in the same town and share custody of the children. She attends her local public school and receives special education services through her Individualized Education Plan (IEP), both during and after school. During the school day, a paraprofessional supports Isabella as ADHD and possible GAD are challenges in her learning environment. While at school she also receives 30 minutes of Cognitive Behavioral Therapy (CBT) twice a week, 30 minutes of Occupational Therapy (OT) twice a week for bladder and bowel issues. She also attends an after school program at her school while her parents work full-time day shifts. During her after school program, Isabella is provided with an hour of music therapy which has helped her attention, anxiety, and behavior.

With the COVID-19 pandemic causing schools to close in March 2020, Isabella is no longer receiving these services and is attending school remotely. Her teachers have been able to create Zoom meetings with the class, her music therapist is creating YouTube videos, and school has sent a packet of worksheets but without the extra aide, Isabella's parents are having a hard time supporting her and the 2 other children while working from home.

Isabella is on a medication to manage her ADHD, but not yet on a medication for GAD (it is pending the psychiatry appointment). Mom and Dad have the medication at both of their houses and are relatively good about reminding her to take it, but sometimes forget to tell each other which has caused accidental missed doses and double doses. Isabella's bowel movements are being tracked by her parents, per advice given by Isabella's PCP, in a notebook - sometimes bowel movements are missed because the notebook is in a different parent's house.

PART 3: EXPLORE THE CAREMAP APP – 10 MINUTES

You are ready to start the Simulation!

BEGIN

Isabella's PCP, Dr. Dorothy, suggested 'Caremap' to help you manage Isabella's health needs and easily communicate updates with Isabella's care providers. You have downloaded the app and are ready to explore!

✓ Begin by visiting the **'My Health'** tab and spend approximately 10 minutes exploring this section of the Caremap App. Please go into each of the sections listed below to a) read the information about Isabella and b) add new information about Isabella as if you were her parent.

Sections to Explore:

- ☐ Snapshot
- ☐ Medical Conditions
- ☐ Medical Equipment
- ☐ High Level Goals
- ☐ Notes
- ☐ Emergency Care
- ☐ Allergies
- ☐ Medications
- ☐ Medical History
- ☐ Hospitalizations
- ☐ Surgeries and Procedures
- ☐ Post Discharge Instructions

Tips

- ➢ *The goal of this section is to familiarize yourself with the Caremap App. Feel free to add or change information.*
- ➢ *Feel welcome to create goals for and information on Isabella. As a reminder, no information that you type into the Caremap app can be seen or shared with the Caremap team.*
- ➢ *Expand and collapse sections by clicking on the title of that section; 'Medical Overview', 'Emergency Care', etc.*
- ➢ *To return back to the previous screen, select the '<' arrow or click 'cancel'.*

- ➢ ***If needed, additional guidance on how to use the Caremap App is available in the Resources Section at the end of this document.***

❖ *In the full version of the app, medical information can be imported directly from the patient's Electronic Medical Record (EMR) and then enhanced with information added by the caregiver.*

PART 4: USE CAREMAP TO COORDINATE ISABELLA'S CARE – 20 MINUTES

Use the Caremap App to help track and coordinate Isabella's care.

1	**MY HEALTH**<br>**You just got off the phone with Dr. Dorothy who, after consulting with a Mental Health Clinician, would like to add a new over-the-counter medication for the constipation Isabella experiences. She would like Isabella to take 17g of a stool softener 'Polyethylene Glycol' on a daily basis.**<br>✓ Add this new medication to Isabella's **'My Health'** section by clicking on any medication in the medication list. On the next screen, click **'Add another'** and add the new prescription: **'17g of Polyethylene Glycol, once per day'** and press **'Save'**.<br><br>**While you are reviewing Isabella's health record, you notice that the date of her last hospitalization is incorrect and it does not include** all **the discharge instructions!**<br>✓ Under **'Medical History'**, click on **'Post Discharge Instructions'**. Update the date of discharge to September 29, 2020. Click into the description and add **"Isabella needs a follow up appointment with GI"** and press **'Save'**.<br><br>**Isabella is struggling with remote learning while she's attending school virtually this year. You want to make a note to check in with her Occupational Therapist (OT) and music therapy teacher to see if there are tools you could use to help Isabella focus.**<br>✓ Click on the **'Notes'** field that sits within the **'Medical Overview'** section. Write yourself a note to follow-up with them next Monday.
2	**TRACK**<br>**At Isabella's last appointment, Dr. Dorothy asked that between now and her next appointment, you track symptoms relative to her physical and behavioral health so they have a better understanding of her day-to-day behaviors. She thinks this information will be especially helpful for Isabella's GI issues and upcoming Psychiatric appointment.**<br>✓ Click the **'Track'** tab and select the 4 goals Dr. Dorothy would like you to track **(urination, mood, bowel movements and sleep)** by clicking on the icon and click **'Save'**.<br>✓ Select one goal at a time to enter information to track Isabella's health for the past three days from today's date.<br>✓ Track a different day or week by clicking the calendar at the top of the screen and selecting the day you would like to track.<br>➢ *If desired, keep adding in new data to see a full report in the **'Insights'** tab. Caremap App can visualize up to 7 days of data at a time.*<br><br>**Urination**<br>✓ Select how many times Isabella urinated three days ago and click **'Next'**<br>✓ Answer **'yes'** or **'no'** to the next question and click **'Next'**<br>✓ Add comments and click **'Done'** or click **'Skip'** if no comments are needed<br>✓ Repeat entering data for yesterday and today<br><br>Continue on next page…

	**Mood**<br>✓ Select the emoji that best represents how Isabella was feeling three days ago and then click **'Next'**<br>✓ Add comments and click **'Done'** or click **'Skip'** if no comments are needed<br>✓ When you are tracking, choose **'down'** or **'at my lowest'** at least once to see an option to contact Isabella's care team to follow-up!<br>✓ Repeat for yesterday and today<br><br>**Bowel Movements**<br>✓ Input how many bowel movements Isabella had three days ago<br>✓ Click **'next'**, add a note if you would like, then click **'Done'**<br>✓ Click **'Touch here to upload image of your child's stool'** to see how you can include pictures<br>✓ Today you will **not** be uploading pictures so click **'Done'**<br>✓ Repeat for yesterday and today<br><br>**Sleep**<br>✓ Enter how long Isabella slept three days ago and click **'Next'**<br>✓ Select the quality of sleep and click **'Next'**<br>✓ Repeat for yesterday and today
3	**TRACK**<br>**When you spoke to Dr. Dorothy about giving Isabella stool softener on a daily basis, she asked that you track when the medication is administered in the Caremap app.**<br>✓ Add medication tracking to the **'Track'** tab by clicking the + option on the top-right of your screen and selecting **'Add your own'** (scroll down to see 'Available' icons). Enter the name **''Polyethylene Glycol'** and choose Type **'Yes or No'** then click **'Save'**. After you create the option to track medication, click **'Save'** in the top-right of the screen and click option **' Polyethylene Glycol'**, to track Isabella's medication use.<br><br>❖ *In the full version of Caremap, Isabella's providers will be able to view this information and provide feedback as needed.*
4	**INSIGHTS**<br>**Dr. Dorothy would like you to check-in after a week of tracking to let her know how Isabella is doing, especially after being on her new medication.**<br>✓ Click the **'Insights'** tab to view the charts and trackers that were created from the data you entered in the **'Track'** tab.<br><br>❖ *In the full version, you will be able to send this report to Dr. Dorothy by clicking **'Share Care Insights'** which creates a clinical summary that can be printed or shared securely. Today you are not sharing the information so proceed to the next step.*
5	**CONNECT**<br>**You want to send Isabella's information to Dr. Dorothy, but you don't have her contact information saved yet!**<br>✓ Click the **'Connect'** tab and add contact information for her PCP. Click the + option in the top-right of your screen and enter the fictional contact information below and click **'Save'**.<br>*First Name: Dorothy*<br>*Last Name: Zbornak*<br>*Relationship: Physician*<br>*Phone Number: 111-222-3333*

	✓ Click on **'Dr. Zbornak'** now listed in the Care Team to see her contact information displayed.
6	**MORE**<br>✓ Click the **'More'** option to find 'Patient Resources', 'Frequently Asked Questions', and more information
7	**SHARE**<br>**Isabella is starting with a new Occupational Therapist (OT) next week since her previous OT was school-based and Isabella is now learning remotely. The plan is for Isabella to meet with the OT twice a week; one session will be virtual and one will be in her Dad's backyard so they can benefit from in-person sessions, but also decrease the coronavirus transmission risk. The new OT would like you to send him Isabella's health summary so he can learn about Isabella before the first session and complete his intake paperwork.**<br>✓ Click the **'My Health'** tab and click **'Share'** in the top left of the screen. Read the prompt and click **'Ok'**. On the next page you will review Isabella's health summary, medical needs, and current goals. Click "Share Now' and then 'Ok'. You have reached the end of Part 4.<br>❖ *In the full version, you will be able to send this report to the contacts in the 'Connect' tab.*

PART 5: FEEDBACK SURVEY – 20 Minutes

We want to hear your feedback! Please fill out the feedback survey to complete the simulation. After the survey is completed, you will be sent a $100 electronic gift card for your participation.

You will find the custom link to the feedback survey in the email sent after you enrolled.

The email is titled "Caremap Simulation Enrollment" and sent from integratedcare@childrens.harvard.edu

Caremap Resources

Please see additional guidance for exploring the functionality in the 'My Health' Section of the Caremap App. This section is <u>not</u> part of the Simulation.

- Under 'Medical Overview', click 'Snapshot' to read an overview of Isabella, written by her parents. To add additional information, click into the text box under 'Describe about you/your child in 2-3 sentences', write in a description, click 'Done', and then scroll to the bottom of the screen to click 'Save'.
- Click 'Medical Conditions' to review Isabella's conditions that were inputted from her EMR. To add additional medical conditions, click 'Add Another', click in to the text box that says 'Condition Name', type in a condition, click 'Done', and click 'Save'.
- Click 'Medical Equipment' and review the equipment Isabella uses. To add additional equipment, click 'Add Another', click into the text box that says 'Equipment Name', add additional items and click 'Save'.
- Click 'High Level Goals' and review Isabella's goal list that was inputted from her EMR. To add additional goals, click 'Add Another', click into the text box that says 'Goal', type in an additional goal and click 'Save'.
- Click the 'Notes' option and then click into the text box that says 'Details' to add a note regarding Isabella's care. Click on 'Reminder Date' to choose a specific date, click 'Done', and then click 'Save'.
- Under 'Emergency Care' click the information below to review what Isabella should do in the event of emergencies. To add additional information, click 'Add another', click the text box that says 'Topic', enter the information then click into the text box below that says 'Description', enter information and click 'Save'.
- Under 'Allergies', click on one of Isabella's allergies to review her allergy list that was inputted from her EMR. To add an additional allergy, click 'Add Another', click into the text box that says 'Allergy' to type in the allergy name, click 'Severity' and choose one of the options listed, click on the textbox below 'Severity' to include more information. Click 'Save'.
- Under 'Medications', click on one of Isabella's medications to review the medication list that was inputted from her EMR. To add an additional medication, click 'Add Another', click into the text box that says 'Medication' to type in the medication name, click 'Dosage' to include that information, and click 'Save'.
- Under 'Medical History', click 'Hospitalizations' to review Isabella's hospitalization admissions, discharges, and more information. To add additional hospitalizations, click 'Add Another', click on date of admission to choose a date and click 'Done'. Click on 'Date of discharge' to choose that date and click 'Done'. Click into the textbox that says 'Description' to add in additional information. 'Remove' or 'Save' the note by clicking those options.
- Click 'Surgeries & Procedures' to review Isabella's procedure and surgery history. To add additional procedures, click 'Date of procedure/surgery' to choose a date and click 'Done'. Click into 'Procedure/surgery details' to enter more information and click 'Save'.
- Click 'Discharge Instructions' to review the discharge instructions from Isabella's previous hospitalizations. To add additional discharge instructions, click 'Add Another' then 'Date of discharge' to choose a date and click 'Done'. Click into the text box below that says 'Discharge Instructions' to type in additional information then click 'Save'.

This is a Multimedia Appendix to a full manuscript published in the J Med Internet Res. For full copyright and citation information see http://dx.doi.org/10.2196/jmir.43933
