# Supplementary material for: Outcomes of End-User Testing of a Care Coordination Mobile App With Families of Children With Special Health Care Needs: Simulation Study
Source: JMIR Form Res. 2023 Aug 28;7:e43993. doi: 10.2196/43993 (PMC10495855; doi:10.2196/43993)
Supplement: Multimedia Appendix 3 [file formative_v7i1e43993_app3.pdf]

Multimedia Appendix: Adapted PICS and uMARS Questionnaire

Caremap Simulation Feedback Survey

Introduction

Please help us understand the experience of using the Caremap app by completing the following survey.

This survey has a total of **29** questions and takes approximately **30** minutes to complete. It is divided into two parts:

- Part A: Experience of Care Integration – Questions to understand your perception of Isabella's family's experience of planning, managing, and tracking Isabella's care when using the Caremap App.
- Part B: Functionality Assessment – Questions to gauge your thoughts on the quality of the Caremap app in terms of design, functionality, usability, etc.

Upon completion of the survey, you will have reached the end of the Simulation. You will then be prompted to enter your e-mail address which will initiate the process for sending you the $100 reward for providing your feedback. Thank you again for your time and effort.

Part A: Experience of Care Integration

For the following questions, please think of the "care team" as Isabella, her family, her doctors, and all the people providing care to Isabella.

1. Based on your experience using Caremap, how easy or difficult would it be for Isabella's family to communicate with her care team members if they had a concern about Isabella's health or care? (Check ONE box)
   - Very difficult
   - Difficult
   - Neither easy nor difficult
   - Easy
   - Very easy
   - I don't know

2. Based on your experience using Caremap, how easy or difficult would it be for Isabella's family to communicate with her care team members about advice they received from Isabella's other care team members? (Check ONE box)
   - Very difficult
   - Difficult
   - Neither easy nor difficult
   - Easy
   - Very easy
   - I don't know

3. Based on your experience using Caremap, how easy or difficult would it be for Isabella's family to know who was responsible for different parts of Isabella's care? (Check ONE box)
   - Very difficult
   - Difficult
   - Neither easy nor difficult
   - Easy
   - Very easy
   - I don't know

4. Based on your experience using Caremap, how easy or difficult would it be for Isabella's family to communicate with her care team members about tests and evaluations that Isabella had recently in order to avoid unnecessary testing or miscommunication? (Check ONE box)
   - Very difficult
   - Difficult
   - Neither easy nor difficult
   - Easy
   - Very easy
   - I don't know

5. Based on your experience using Caremap, how easy or difficult would it be for Isabella's family to track that her care team members were following through with their responsibilities related to Isabella's care? (Check ONE box)
   - Very difficult
   - Difficult
   - Neither easy nor difficult
   - Easy
   - Very easy
   - I don't know

6. Based on your experience using Caremap, how easy or difficult would it be for Isabella's family to communicate with her care team members about challenges that make it hard for them to manage Isabella's health? (Check ONE box)
   - Very difficult
   - Difficult
   - Neither easy nor difficult
   - Easy
   - Very easy
   - I don't know

7. Based on your experience using Caremap, how easy or difficult would it be for Isabella's family to communicate with her care team members about challenges in their family's life that causes them stress because of Isabella's health or care needs? (Check ONE box)
   - Very difficult

- Difficult
- Neither easy nor difficult
- Easy
- Very easy
- I don’t know

8. Based on your experience using Caremap, how easy or difficult would it be for Isabella’s family to coordinate visits and treatments with her care team members? (Check ONE box)
   - Very difficult
   - Difficult
   - Neither easy nor difficult
   - Easy
   - Very easy
   - I don’t know

9. Based on your experience using Caremap, how easy or difficult would it be for Isabella’s family to track progress toward Isabella’s short-term care goals,? (Check ONE box)
   - Very difficult
   - Difficult
   - Neither easy nor difficult
   - Easy
   - Very easy
   - I don’t know

10. Based on your experience using Caremap, how easy or difficult would it be for Isabella’s family to track progress toward Isabella’s long-term care goals, meaning goals 6 months or longer into the future? (Check ONE box)
    - Very difficult
    - Difficult
    - Neither easy nor difficult
    - Easy
    - Very easy
    - I don’t know

11. Based on your experience using Caremap, how easy or difficult would it be for Isabella’s family to think about care needs that Isabella might have in the future and to make sure that they are taken care of instead of waiting until there is an actual problem? (Check ONE box)
    - Very difficult
    - Difficult
    - Neither easy nor difficult
    - Easy

    Very easy

- I don't know

12. Based on your experience using Caremap, how easy or difficult would it be for Isabella's family to make sure that the "big picture" was taken into account when decisions and recommendations were made about Isabella's care? (Check ONE box)
    - Very difficult
    - Difficult
    - Neither easy nor difficult
    - Easy
    - Very easy
    - I don't know

Part B: Functionality Assessment

1. Is the Caremap app interesting to use? (Check ONE box)
    - Not interesting at all
    - Mostly uninteresting
    - OK, neither interesting nor uninteresting; would engage a user for a brief time (< 5 minutes)
    - Moderately interesting; would engage a user for some time (5-10 minutes total)
    - Very interesting, would engage a user in repeat use

2. Does Caremap allow user input, provide feedback, contain prompts (reminders, sharing options, notifications, etc.)? (Check ONE box)
    - No interactive features and/or no response to user input
    - Some, but not enough interactive features which limits app's functions
    - Basic interactive features to function adequately
    - Offers a variety of interactive features, feedback and user input options
    - Very high level of responsiveness through interactive features, feedback and user input options

3. Is the Caremap app content (visuals, language, design) appropriate for families/caregivers? (Check ONE box)
    - Completely inappropriate, unclear or confusing
    - Mostly inappropriate, unclear or confusing
    - Acceptable but not specifically designed for families/caregivers. May be inappropriate/unclear/confusing at times
    - Designed for families/caregivers, with minor issues
    - Designed specifically for families/caregivers, no issues found

4. How easy is it to learn how to use the Caremap app; how clear are the menu labels, icons and instructions? (Check ONE box)
    - No/limited instructions; menu labels, icons are confusing; complicated
    - Takes a lot of time or effort
    - Takes some time or effort

- Easy to learn (or has clear instructions)
- Able to use app immediately; intuitive; simple (no instructions needed)

5. Does moving through Caremap make sense; Does the app have all necessary links between screens? (Check ONE box)
   - No logical connection between screens at all /navigation is difficult
   - Understandable after a lot of time/effort
   - Understandable after some time/effort
   - Easy to understand/navigate
   - Perfectly logical, easy, clear and intuitive screen flow throughout, and/or has shortcuts

6. Is the arrangement and size of buttons, icons, menus and content on the Caremap screen appropriate? (Check ONE box)
   - Very bad design, cluttered, confusing
   - Bad design, random, unclear, some options difficult to select/locate/see/read
   - Satisfactory, few problems with selecting/locating/seeing/reading items
   - Mostly clear, able to select/locate/see/read items
   - Professional, simple, clear, orderly, logically organized

7. How good does the Caremap app look? (Check ONE box)
   - Ugly, unpleasant to look at, poorly designed, clashing, mismatched colours
   - Bad – poorly designed, bad use of colour, visually boring
   - OK – average, neither pleasant, nor unpleasant
   - Pleasant – seamless graphics – consistent and professionally designed
   - Beautiful – very attractive, memorable, stands out; use of colour enhances app features/menu

8. Is the app content in Caremap accurate, well written, and relevant to the goal/topic of the app? (Check ONE box)
   - N/A- There is no information within the app
   - Irrelevant/inappropriate/incoherentnot accurate
   - Poor. Barely relevant/appropriate/coherentaccurate
   - Moderately relevant/appropriate/coherentaccurate
   - Relevant/appropriate/coherent/accurate
   - Highly relevant, appropriate, coherent, and accurate

9. Is the visual explanation of concepts in the Caremap app (charts/graphs/images/videos, etc.) clear, logical, accurate? (Check ONE box)
   - N/A- There is no visual information within the app (e.g. it only contains audio, or text)
   - Completely unclear/confusing/wrong or necessary but missing
   - Mostly unclear/confusing/wrong
   - OK but often unclear/confusing/wrong
   - Mostly clear/logical/correct with negligible issues
   - Perfectly clear/logical/accurate

10. Does the information within the Caremap app seem to come from a credible source? (Check ONE box)
    - N/A- There is no information within the app
    - Suspicious source
    - Lacks credibility
    - Not suspicious but legitimacy of source is unclear
    - Possibly comes from a legitimate source
    - Definitely comes from a legitimate/specialized source

11. Would you recommend the Caremap app to people who might benefit from it? (Check ONE box)
    - Not at all I would not recommend this app to anyone
    - There are very few people I would recommend this app to
    - Maybe – there are several people I would recommend this app to
    - There are many people I would recommend this app to
    - Definitely, I would recommend this app to everyone

12. How many times do you think you would use this app in the next 12 months if it was relevant to you? (Check ONE box)
    - None
    - 1-2
    - 3-10
    - 10-50
    - >50

13. Would you pay for this app? (Check ONE box)
    a. 1 Definitely not
    b. 2
    c. 3
    d. 4
    e. 5 Definitely yes

14. This app has increased my knowledge/understanding of what's possible when it comes to coordinating my child's care across different members of the care team. (Check ONE box)
    a. 1 Strongly disagree
    b. 2
    c. 3
    d. 4
    e. 5 Strongly Agree

    Please elaborate (optional):
    [FREE TEXT RESPONSE]

15. The app would make me more confident at coordinating my child's care across the different members of the care team. (Check ONE box)
    a. 1 Strongly disagree
    b. 2
    c. 3
    d. 4
    e. 5 Strongly Agree

    Please elaborate (optional):
    [FREE TEXT RESPONSE]

16. Use of this app would improve my ability to coordinate my child's care. (Check ONE box)
    a. 1 Strongly disagree
    b. 2
    c. 3
    d. 4
    e. 5 Strongly Agree

    Please elaborate (optional):
    [FREE TEXT RESPONSE]

17. What is your overall (star) rating of the Caremap app? (Check ONE box)

1	★	One of the worst apps I've used
2	★★	
3	★★★	Average
4	★★★★	
5	★★★★★	One of the best apps I've used

This is a Multimedia Appendix to a full manuscript published in the J Med Internet Res. For full copyright and citation information see http://dx.doi.org/10.2196/jmir.43933
